# Enhancing the accumulation of linoleic acid and α-linolenic acid through the pre-harvest ethylene treatment in *Camellia oleifera*

**DOI:** 10.3389/fpls.2023.1080946

**Published:** 2023-02-24

**Authors:** Hongbo Li, Xiaoling Ma, Weiqi Wang, Jiaxi Zhang, Yuanzhe Liu, Deyi Yuan

**Affiliations:** Key Laboratory of Cultivation and Protection for Non-Wood Forest Trees of the Ministry of Education, Central South University of Forestry and Technology, Changsha, China

**Keywords:** *C. oleifera*, linoleic acid, α-linolenic acid, ethylene, transcriptome, regulation

## Abstract

*Camellia oleifera* Abel. (*C. oleifera*) is an important woody edible oil tree species in China. The quality of *C. oleifera* oil (tea oil) is mainly determined by the contents of linoleic acid (LA) and α-linolenic acid (ALA). However, how to increase the contents of LA and ALA in tea oil and the corresponding regulating mechanism have not been clarified. In the present study, we found that the LA and ALA contents in *C. oleifera* seeds were significant positively associated with the concentrations of ethephon and were decreased by ethylene inhibitor treatment. Furthermore, 1.5 g L^-1^ ethephon could receive an optimal LA and ALA contents without adverse effects to the growth of ‘Huashuo’ trees in this study. The ethephon treatment also increased the contents of 1-aminocyclopropane-1-carboxylic acid (ACC), sucrose, soluble sugar and reducing sugar contents in seeds. Transcriptome analysis further suggested that exogenous ethephon application enhanced the accumulation of LA and ALA *via* regulating genes involved in LA and ALA metabolism, plant hormone signal transduction pathways, and starch and sucrose metabolism. Our findings confirm the role of ethylene in LA and ALA regulation and provide new insights into the potential utilization of ethylene as a LA and ALA inducer in *C. oleifera* cultivation.

## Introduction

*Camellia oleifera* Abel. (*C. oleifera*), a member of the genus *Camellia* in the tea family (Theaceae), has been cultivated in China for more than 2300 years (Wu et al., 2020; Song et al., 2022). As one of the world’s four major woody oil trees, together with oil palm, olive and coconut, it has been widely used as edible oil, lubricant, and cosmetics (Gong et al., 2020; Lu et al., 2022). With the rapid development of *C. oleifera* industry, the cultivation area of *C. oleifera* forest is constantly expanding, and the yield of fruits is also growing (Wu et al., 2020). The postharvest *C. oleifera* fruits need to peel the shells before extracting oil. The main product of *C. oleifera* seeds, used to extract edible vegetable oil. The by-products of *C. oleifera*, such as tea meal and fruit shells, can be processed into important chemicals and other industrial raw materials to manufacture soap, green fertilizer, activated carbon, and so forth (Xing et al., 2011; Lu et al., 2021). *C. oleifera* oil (tea oil) is commonly known as ‘eastern olive oil’, because more than 90.0% components are unsaturated fatty acids (Wu et al., 2020). Further, the oleic acid (OA) content accounts for fatty acids more than 80.0%, but the contents of linoleic acid (ω-6 fatty acid, LA) and α-linolenic acid (ω-3 fatty acid, ALA) which are essential for human body are only approximately 8.0% and 0.2%, respectively (Gong et al., 2020; Lu et al., 2021; Zhang et al., 2021). Therefore, increasing the LA and ALA contents in tea oil can potentially improve the oil quality.

Previous studies have focused on a series of genes vital to LA and ALA synthesis. Fatty acid desaturases (FADs) are specific enzymes, which are responsible for the conversion of OA to LA and ALA successively (Yang et al., 2020). The microsomal delta-12*FAD2* genes encode enzymes involved in the conversion of OA to LA in fatty acid biosynthesis pathway. The expression level of *FAD2* was negatively correlated with the content of OA in hickory (Huang et al., 2016) and olive (Unver et al., 2017). Antisense expression of the *FAD2* gene in *Brassica juncea* increased the content of OA compared with that in the parental line (Indira et al., 2004). Previous studies have shown that the genetic improvement was achieved by silencing the *FAD2* gene to increase OA and simultaneously to reduce LA in soybean (Yang et al., 2017), canola (Stoutjesdijk et al., 2000), groundnut (Yin et al., 2007), and linseed (Chen et al., 2015). *Lipoxygenase* (*LOX*) is a key enzyme gene that regulates the first step in the conversion of LA into other substances. The high expression of *LOX* in *C. oleifera* seeds at maturity stage is not conducive to the accumulation of ALA and the formation of excellent quality of *C. oleifera* (Nan et al., 2014). However, the understanding of the molecular regulation of fatty acid composition during seed development of *C. oleifera* is still limited.

Plant hormones are naturally produced by plant metabolism and play key roles in plant growth, development, and stress adaptations (Li and Li, 2019; Li et al., 2020; Song et al., 2022). Thus, exploring the application of plant hormones is conductive to increasing the LA and ALA contents and improving the tea oil’s quality. Ethylene is involved in the regulation of multiple biological processes, such as fruit ripening, flower and leaf senescence, and fruit abscission (Vandenbussche et al., 2012; Zhou et al., 2015; Xin et al., 2019). Ethephon (2-chloroethylphosphonic acid), an ethylene-releasing molecule, is widely used as an artificial ripening agent in many fruit crops (Hu et al., 2021). For example, external application of ethephon has been proposed to increase ripening rate and facilitate early harvesting in date fruits (*Phoenix dactylifera* L.) (Al-Saif et al., 2017). In pace with the effect of enhancing fruit ripening, ethylene also can generate husk dehiscence (Sriyook et al., 1994).

Researchers have found a positive correlation between fruit ripening and oil content (Lin et al., 2018; Zhang et al., 2021). The mature seeds have a higher ratio of unsaturated fatty acid content to saturated fatty acid content and a higher ratio of monounsaturated fatty acid content to polyunsaturated fatty acid content relative to the immature seeds (Lin et al., 2018). Moreover, several studies have addressed that ethylene can regulate the aroma production (Gunther et al., 2015; Zhang et al., 2019; Zhang et al., 2021). As the aroma is synthesized by LOX system with fatty acids as major precursors (Zhou et al., 2015; Zhang et al., 2018), this seems the ethylene might have a regulating effect on unsaturated fatty acid content. However, study on the relationship between ethylene and the synthesis of LA and ALA is largely unknown.

In the present study, the potential role of ethylene on fatty acid composition in *C. oleifera* seeds was investigated. Exogenous ethylene significantly promoted the accumulation of LA and ALA contents in *C. oleifera* seeds, whereas ethylene inhibitor (aminooxyacetic acid, AOAA) reduced the accumulation of LA and ALA contents. The 1.5 g L^-1^ ethephon treatment of ‘Huashuo’ trees could receive an optimal LA and ALA contents without affecting leaf growth and fruit phenotypic traits. The ethephon treatments also increase the contents of 1-aminocyclopropane-1-carboxylic acid (ACC), sucrose, soluble sugar and reducing sugar contents in seeds. Transcriptome analysis further showed that ethylene can enhance the accumulation of LA and ALA by regulating genes including LA and ALA metabolism, plant hormone signal transduction pathways, and starch and sucrose metabolism. These results demonstrate the role of ethylene in LA and ALA regulation and provide new insights into the potential utilization of ethylene as a LA and ALA inducer in *C. oleifera* cultivation.

## Materials and methods

### Plant materials, treatments, and fruit phenotypic trait analysis

*C. oleifera* ‘Huashuo’, which was grown at the experimental stations of the Central South University of Forestry and Technology located in Wangcheng and Liuyang of Hunan Province, was used in this study. At the early stage of oil synthesis (August 22, 2020), the trees were treated with water (the control), 1.5 g L^-1^ ethephon (ETH), and 4 mM AOAA to assess the effect of ethylene on the oil and relative fatty acid contents in the mature seeds. The concentrations of ethephon and AOAA were selected based on our previous experiments (> 1.5 g L^-1^ ethephon, or > 4 mM AOAA have adverse effects to the growth of ‘Huashuo’ trees). In addition, uncracking and cracking fruits under the natural conditions were collected on August 22, 2020, which were used to analyze the ACC content and relative fatty acid contents.

To further judge ethylene’s effects on the oil and relative fatty acid contents, ethephon treatment experiments with different concentrations were conducted in Liuyang at the late stage of oil synthesis of ‘Huashuo’ trees (October 30, 2020). The trees were treated with 0 (the control), 0.5 (ETH1), 1.0 (ETH2), and 1.5 g L^-1^ (ETH3) ethephon, respectively. The fruits and leaves were randomly collected from four directions of trees at the same day of fruit mature stage (one week after treatment) as previously described (Song et al., 2021; Zhang et al., 2021). Fifteen fresh fruits, which were divided into three groups (five fruits each group), were randomly collected in each treatment of ‘Huashuo’ and measured fruit phenotypic trait indexes as previously described (Song et al., 2021). Subsequently, seeds and shells were immediately separated from the fruits. The seeds were mixed evenly and divided into two sections. One part was frozen in liquid nitrogen and stored at −80°C for physiological index determination, ACC content analysis, and RNA extraction. The other part was dried to measure the oil content and fatty acid composition. The separated shells were divided into two sections. One was fixed in 2.5% (v/v) glutaraldehyde solution for scanning electron microscopy (SEM) as previously described (Hu et al., 2021). The other was frozen in liquid nitrogen and stored at −80°C for RNA extraction. The leaves were randomly selected to measure chlorophyll and carotenoid contents. Moreover, ethephon treatment experiments with different concentrations were repeated in Liuyang on October 30, 2021, to verify the effect of ethephon on the oil and relative fatty acid contents.

‘Xianglin 210’ planted in Liuyang was also used to verify the effects of ethephon on the oil, relative fatty acid contents and the growth of *C. oleifera*. Due to the differences of the fruit mature stages between the cultivars ‘Huashuo’ and ‘Xianglin 210’ (Zhang et al., 2021), the treatment time was uniformly selected at the late stage of oil synthesis (one week before harvest). The trees were treated with 0 (the control), 1.5 (ETH3), 2.0 (ETH4), and 2.5 g L^-1^ (ETH5) ethephon on October 20, 2020.

*C. oleifera* trees with similar growth conditions and potentials and without diseases were randomly selected in this study. Each treatment has three trees in this study. Treatments were sprayed to run-off with 0.05% Tween 80 solution using a motorized backpack sprayer according to a previously described method (Hu et al., 2021). Weights of fresh fruits, dry seeds, and fresh seeds were measured using a CS5000 electronic balance (Ohaus International Trading Co., Ltd., Shanghai, China). The fruit transverse diameter, fruit longitudinal diameter, and shell thickness were measured using the minimum resolution of 0.01 mm with a Vernier caliper (Mitutoyo Co., Ltd., Kanagawa Prefecture, Kawasaki, Japan). The fruit shape index = fruit longitudinal diameter/fruit transverse diameter, the fresh seed ratio = (fresh seed weight/fresh fruit weight) × 100% and moisture content = ((fresh seed weight-dry seed weight)/fresh seed weight) × 100%. The shell cracking rate was calculated according to the following formula: shell cracking rate = (number of cracked fruits/number of total fruits) × 100%. All data on fruit phenotypic traits of mature stage in this work are presented as the mean ± SD of three biological replicates.

### Oil content and relative fatty acid content determination

Fresh *C. oleifera* seeds collected at different treatment groups were dried in an oven at 60°C to a constant weight. All dried seeds were powdered by a mill (FOSS Scino (Suzhou) Co Ltd., Suzhou, China), and 5 g of three independent samples of dry seeds were weighed. Total tea oil was extracted according to the manufacturer’s protocol (Gong et al., 2020). Approximately 50 mL of petroleum ether was added to the aluminum cup in Soxtec device for extraction, and the machine temperature was set to 75°C. The program was as follows: 30 minutes of digestion, 150 minutes of extraction, and 60 minutes of solvent evaporation and recovery. The extracted oil is stored in the centrifuge tube for further use. Oil content was calculated according to the following formula: Oil content = [(weight of filter paper bag before extraction − weight of filter paper bag after extraction)/weight of powder] × 100%. Each sample had three biological replicates, and all oil contents in this study are performed with three biological replicates.

Fatty acid composition was determined using gas chromatography (Shimadzu GC-2014, Shimadzu, Kyoto, Japan) following the manufacturer’s instructions (Gong et al., 2020). Each sample had three biological replicates. Fatty acid methyl esters were prepared using NaOH/methanol method. A total of 60 mg of oil was transferred into a ground glass stoppered test tube, dissolved by 4 mL isooctane. Internal standard solution (triglyceride undecanoate, 100 μL) and 200 μL of potassium hydroxide methanol solution were added to the sample. The samples were mixed for 30 s on vortex mixer, and then allowed to clarify. One gram of sodium bisulfate was added, and the test tube was shaken vigorously to neutralize the potassium hydroxide. After the salt settled, the upper layer solution was used for chromatographic analysis. The gas chromatograph program was as follows: flame ionization detector temperature, 250°C; sample inlet temperature, 250°C; chromatographic column, 60 cm × 0.25 mm × 0.2 μm; carrier gas, nitrogen; split ratio, 1:50; sample injection volume, 1 μL; heating process, 50°C (2 min), 170°C (10°C/min, stored for 10 min), 180°C (2°C/min, stored for 10 min), and 220°C (4°C/min, stored for 22 min).

### Scanning electron microscopy observation

Shells of ETH3 and control were collected at the fruit mature stage of ‘Huashuo’. The samples were fixed in 2.5% (v/v) glutaraldehyde solution for 3 h, and then post-fixed in 1.0% (w/v) osmium tetroxide for 2 h. They were washed in 0.1 mol^-1^ sodium phosphate buffer. Dehydration was completed in a graded series of ethanol. Anhydrous ethanol was replaced with 3-methylbutyl acetate for SEM. The samples were critical-point dried and sputter-coated with gold, and the fracture plane of different samples was observed using SEM (Zeiss Supra 10 vp; Carl Zeiss Microscope, NY, USA) at 50-fold magnification (20 kV).

### Determination of physiological indicators, endogenous ethylene content, and leaf chlorophyll and carotenoid contents in ‘Huashuo’ seeds

Seeds of ETH3 and control were collected at the fruit mature stage of ‘Huashuo’. The soluble sugar content was determined using anthrone colorimetry (Liu et al., 2015). The contents of sucrose and reducing sugars were evaluated using the 3,5-dinitrosalicylic acid method (Yang et al., 2017). Endogenous ethylene content was evaluated by the ACC content (Hu et al., 2021). The grinded samples of 0.5 g were homogenized in phosphate-buffered saline, and then centrifuged at for 20 min (4°C, 12000 rpm). These supernatants were used to measure the ACC contents. The ACC contents of the seed and shell were measured according to the Plant 1-aminocyclopropane carboxylic acid ACC kit (Shanghai Jingkang Bioengineering, Co., Ltd., Shanghai, China) instructions (Hu et al., 2021). The OD450 value was determined using a microplate reader (BioTek, Winooski, Vermont, USA).

Ten leaves from one tree were randomly selected to measure the chlorophyll content for each biological replicate. Leaves of ethephon treatment and control were cut into filaments. The filaments of 0.2 g were immersed in an acetone–ethanol mixture (2:1, v/v) for 24 h (4°C, darkness). The samples were shaken several times during the experiment. The absorbance indexes at 663 and 645 nm of the solution were assessed by a spectrophotometer (UV-1100, Mapada, China). The chlorophyll a and chlorophyll b contents were calculated, referring to the method of Zhang et al. (Zhang et al., 2021).

### RNA-seq and data analysis

Total RNA was isolated from shells of ETH3 and control in ‘Huashuo’ at the fruit mature stage from field grown plants using the Trizol Reagent Kit (Invitrogen, Carlsbad, USA). The quality of total RNA was evaluated using an Agilent 2100 Bioanalyzer (Agilent Technologies, Palo Alto, USA). The concentration and purity of each mRNA sample was determined using NanoDrop ND-1000 spectrophotometer (NanoDrop Technologies, Wilmington, DE, USA). The construction of the libraries and the RNA-seq were performed by the Biomarker Technologies Co., Ltd (Beijing, China). After removing the adaptor sequences and low-quality reads, high quality clean reads from all samples were assembled using Trinity software (release-2012-10-05) to construct unique consensus sequences for reference (Chen et al., 2019). These sequences obtained from the trinity assembly were called unigenes. These unigenes were annotated using the BLASTx alignment (*E*-value ≤ 10^-5^) to various public databases (the NCBI nonredundant protein (Nr) database, Kyoto Encyclopedia of Genes and Genomes (KEGG) database, Clusters of Orthologous Group (COG), Swiss-Prot protein database, and Gene Ontology (GO) database). The unigenes expression was calculated according to the reads per kilobase transcriptome per million mapped reads (RPKM) method. Genes showing differences in expression between two samples were identified using DESeq2 software (Love et al., 2014). Differentially expressed genes (DEGs) were evaluated based on false discovery rate (FDR < 0.05) and fold change (FC ≥ 2). Furthermore, functional enrichment analyses of DEGs including GO functions and KEGG pathways were implemented.

### Quantitative real-time reverse transcription PCR analysis

To validate the expression patterns of the key genes related to LA and ALA synthesis and regulation, 17 DEGs were selected for qRT-PCR analysis of shells (ETH3 and control) and seeds (ETH1, ETH2, ETH3, and control) during the fruit mature stage in 2020. These genes were selected given their important function and high expression abundance. The qRT-PCR was performed with a Lightcycler 480 (Roche, Basel, Switzerland) using the SYBR Green I Master Kit (Roche). The relative expressions of selected genes were calculated from three biological replicates using the 2^-ΔΔCt^ (Livak and Schmittgen, 2001). *CoEF-1α* was adopted as internal gene (Hu et al., 2021). The specific primers for qRT-PCR are listed in **Table S1**.

### Statistical analysis

All experimental indexes were measured in triplicate biological replicates. Data were shown as means ± SD. Statistical analysis of the means was carried out by one-way ANOVA in the IBM SPSS 20 package for Windows (IBM, New York, NY, USA). Differences of *P* < 0.05 were evaluated statistically significant.

## Results

### The contents of LA and ALA were increased in *C. oleifera* seeds after ethephon treatment

After the treatments of ETH or AOAA at the early stage of oil synthesis of ‘Huashuo’ trees, the mature seeds were collected to measure the oil and relative fatty acid contents in Wangcheng. The results showed that the OA content of ETH declined by 2.6% compared with control (82.8%), but the AOAA showed an increase of 2.9% (**Table 1**). Similarly, the LA and ALA contents increased by 25.5% and 33.3% in ETH, and declined by 25.5% and 20.0% in AOAA, respectively (**Table 1**). We found no differences in contents of oil and Arachidonic acid (AC) under the ETH and AOAA treatments (**Table 1**). Although the contents of Palmitic acid (PA) and Stearic acid (SA) in ETH treatment had significantly affected when compared with the control, there was no definite change patterns among the ETH, control and AOAA (**Table 1**). The opposite change patterns of OA, LA and ALA contents between ethephon and AOAA suggested that the ethylene may influence the synthesis of unsaturated fatty acid.

**Table 1:** Oil and relative fatty acid contents of the treatments and control in ‘Huashuo’.

Location	Treatment time	Treatment	Seed oil content (%)	Oleic acid relative content (%)	Linoleic acid relative content (%)	α-linolenic acid relative content (%)	Arachidonic acid relative content (%)	Palmitic acid relative content (%)	Stearic acid relative content (%)
Wangcheng	2020/8/22	Control	29.75 ± 0.53	82.81 ± 0.02	6.99 ± 0.02	0.30 ± 0.00	0.62 ± 0.02	7.69 ± 0.02	1.58 ± 0.02
		ETH	28.33 ± 0.91	80.64 ± 0.02^**^	8.77 ± 0.04^**^	0.40 ± 0.00^**^	0.59 ± 0.01	8.18 ± 0.01^**^	1.43 ± 0.03^**^
		AOAA	29.45 ± 0.23	85.23 ± 0.02^**^	5.21 ± 0.00^**^	0.24 ± 0.00^**^	0.60 ± 0.00	7.14 ± 0.01	1.58 ± 0.00

Data are represented as the mean values ± standard deviation (SD, n=15). ETH and AOAA represent 1.5 g L^-1^ ethephon and 4 mM aminooxyacetic acid (ethylene inhibitor), respectively. Double asterisks indicate the difference at P < 0.01.

**Figure 1:**
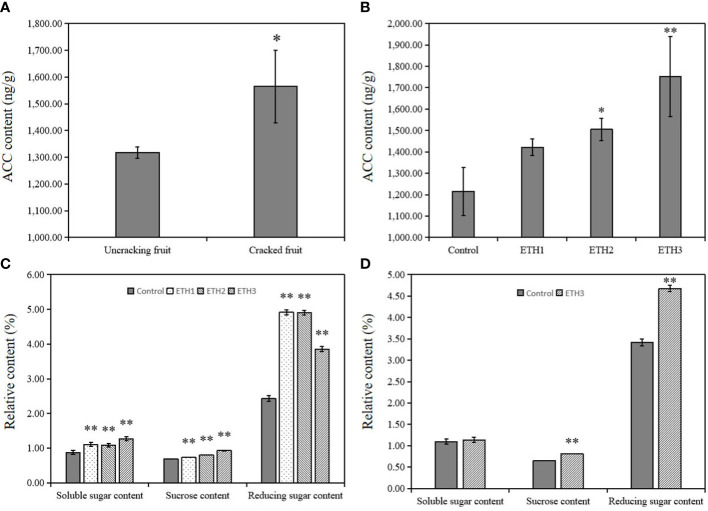
Contents of ACC and sugar in *C.oleifera* ‘Huashuo’ seeds harvested in Liuyang. **(A)** ACC contents in seeds of uncracking and cracking fruits; **(B)** ACC contents of seeds with different treatments (ETH1, ETH2, ETH3), and the control in 2020; **(C)** Sucrose, soluble sugar, and reducing sugar contents of seeds with different treatments (ETH1, ETH2, and ETH3), and the control in 2020; **(D)** Sucrose, soluble sugar, and reducing sugar contents of seeds with ETH3 treatment and the control in 2021. ETH1, ETH2, and ETH3 represent 0.5, 1.0, and 1.5 g L^-1^ ethephon, respectively. Single and double asterisks indicate differences at *P* < 0.05 and *P* < 0.01, respectively.

We also analyzed the ACC and relative fatty acid contents between the fruits of dehiscence and un-dehiscence of ‘Huashuo’ which grew on the natural condition in 2020. The ACC content was significantly higher (*P =* 0.036) in the seeds of cracking fruits, approximately 1.19-fold higher than uncracking fruits (**Figure 1**). Meanwhile, the contents of LA, and ALA in seeds of cracking fruits significantly increased and the OA content was decreased (**Table S2**).

### Screening of appropriate ethephon concentration to increase the contents of LA and ALA without affecting the growth of *C. oleifera*

To gain further insight into the effect of ethylene on the oil and relative fatty acid contents, three ethephon treatments (ETH1, ETH2, and ETH3) and controls of ‘Huashuo’ had been evaluated for two consecutive years (2020 and 2021) in Liuyang. With the gradual increase of ethylene concentration, the contents of LA and ALA also increased gradually. Such as in 2020, when compared with the control, the LA and ALA contents of ETH1, ETH2, and ETH3 increased by 13.6%, 15.5%, 18.0% and 14.3%, 19.1%, 19.1%, respectively (**Table 2**). On the contrary, the OA contents of ETH1, ETH2, and ETH3 in 2020 declined by 0.4%, 0.8%, and 1.0%, respectively (**Table 2**). The consistent results were also received from the ‘Huashuo’ in 2021 (**Table 2**) and ‘Xianglin210’ in 2020 (**Table S3**). However, we also found no effect of ethylene on oil content both in 2020 and 2021 experiments (**Tables 2**, **S3**). These results suggested that application of ethephon has a positive effect on the contents of LA and ALA in *C. oleifera*.

**Table 2:** Oil and relative fatty acid contents of three ethephon treatments and control in ‘Huashuo’ for two consecutive years.

Location	Treatment time	Treatment	Seed oil content (%)	Oleic acid relative content (%)	Linoleic acid relative content (%)	α-linolenic acid relative content (%)	Arachidonic acid relative content (%)	Palmitic acid relative content (%)	Stearic acid relative content (%)
Liuyang	2020/10/30	Control	36.13 ± 0.21	86.78 ± 0.02	3.67 ± 0.02	0.21 ± 0.01	0.58 ± 0.00	6.57 ± 0.02	2.19 ± 0.01
		ETH1	35.41 ± 0.69	86.45 ± 0.25^**^	4.17 ± 0.15^**^	0.24 ± 0.02^**^	0.61 ± 0.02	6.47 ± 0.07^**^	2.06 ± 0.03^**^
		ETH2	35.23 ± 0.29	86.09 ± 0.02^**^	4.24 ± 0.01^**^	0.25 ± 0.00^**^	0.61 ± 0.01	6.51 ± 0.01^*^	2.16 ± 0.00
		ETH3	35.04 ± 0.93	85.92 ± 0.01^**^	4.33 ± 0.00^**^	0.25 ± 0.02^**^	0.60 ± 0.02	6.65 ± 0.01^**^	2.39 ± 0.01^**^
Liuyang	2021/10/30	Control	34.97 ± 0.06	79.33 ± 0.01	9.20 ± 0.01	0.39 ± 0.00	0.57 ± 0.00	9.28 ± 0.01	1.24 ± 0.00
		ETH1	35.73 ± 0.09	78.68 ± 0.14**	10.20 ± 0.07**	0.41 ± 0.01**	0.57 ± 0.02	8.75 ± 0.06**	1.25 ± 0.01
		ETH2	35.17 ± 0.05	77.52 ± 0.02**	10.93 ± 0.02**	0.45 ± 0.00**	0.57 ± 0.01	9.19 ± 0.00*	1.37 ± 0.00**
		ETH3	35.13 ± 0.09	75.02 ± 0.06**	12.49 ± 0.03**	0.48 ± 0.00**	0.55 ± 0.01	10.04 ± 0.02**	1.42 ± 0.01**

Data are represented as the mean values ± standard deviation (SD, n=15). ETH1, ETH2, and ETH3 represent 0.5, 1.0, and 1.5 g L^-1^ ethephon, respectively. Single and double asterisks indicate differences at P < 0.05 and P < 0.01, respectively.

**Figure 2:**
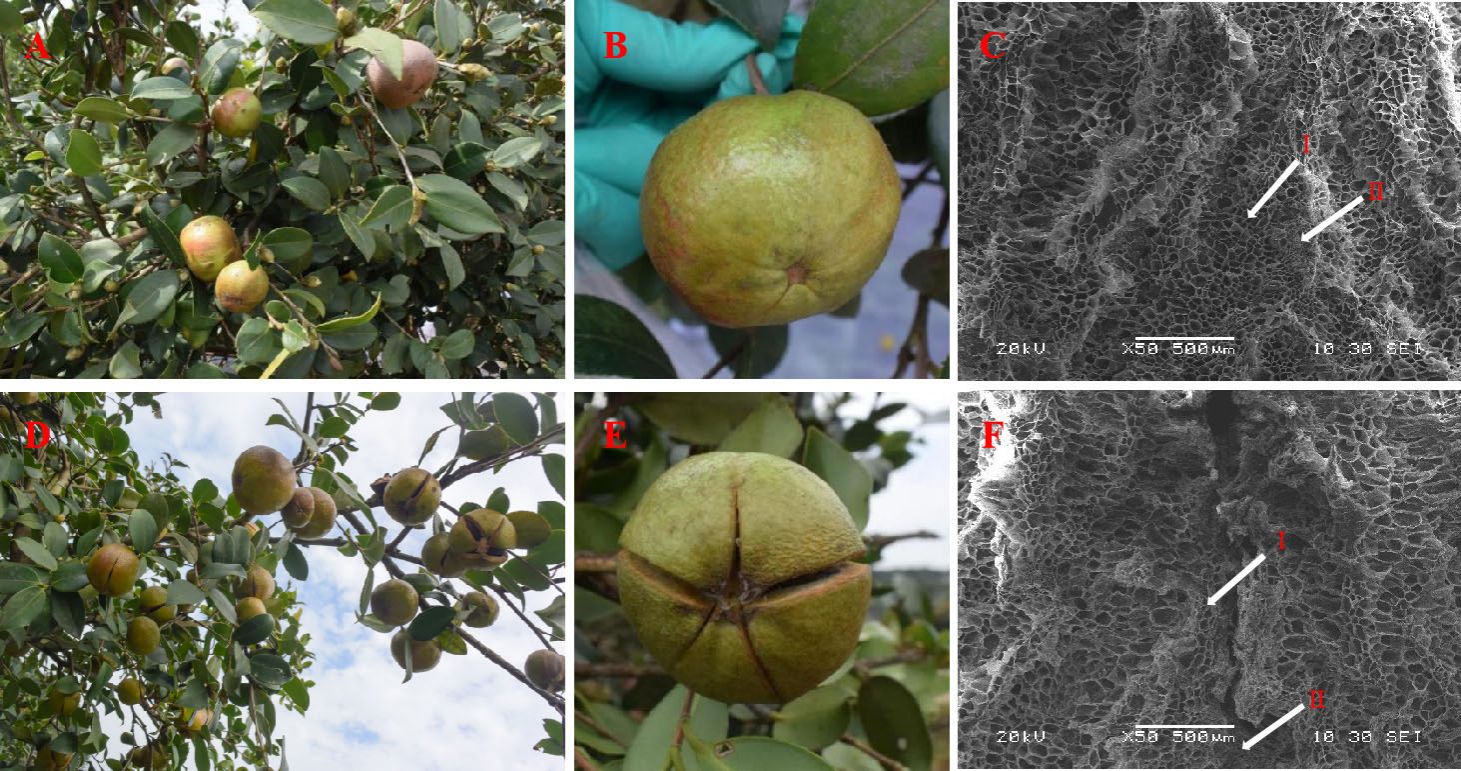
Effects of 1.5 g L^-1^ ethephon treatment on phenotype in fruits and shells of *C. oleifera* ‘Huashuo’. Fruits of *C. oleifera* ‘Huashuo’ after the treatment with the control **(A, B)**; Fruits of ‘Huashuo’ after the treatment with 1.5 g L^-1^ ethephon **(D, E)**; Scanning electron microscopes of the shells structures of the control **(C)** and 1.5 g L^-1^ ethephon treatment **(F)**. The arrows indicate fiber cells (I) and parenchyma cells (II).

To clarify the effects of different concentrations of ethephon on the main characteristics of *C. oleifera* trees and fruits, the growth status, contents of chlorophyll and carotenoid in leaves, and the main fruit traits of ‘Huashuo’ in treatments (ETH1, ETH2, and ETH3) and the control of 2020 were analyzed one week after treatment. We found no evident phenomenon of falling leaves and fruits were observed (**Figures 2A, D**). We also found no significant differences in the contents of chlorophyll a, chlorophyll b, chlorophyll ab, and carotenoid between the treatments and the control (**Figure S1A**). In addition, the main fruit traits also showed no significant differences between the treatments and the control in fresh fruit weight, fruit transverse diameter, fruit longitudinal diameter, fruit shape index, shell thickness, and fresh seed ratio, whereas only the moisture content had a significant reduction in all the ethephon treatments (**Table 3**). These showed that 0.5–1.5 g L^-1^ ethephon did not affect the growth of ‘Huashuo’ trees.

**Table 3:** Fruit phenotypic traits of the three ethephon treatments and the control in ‘Huashuo’.

Treatment	Fresh fruit weight (FW)/g	Fruit transverse diameter/mm	Fruit longitudinal diameter/mm	Fruit shape index	Shell thickness/mm	Fresh seed rate/%	Moisture content (%)	Shell cracking rate/%
Control	54.58 ± 7.66	50.11 ± 2.86	38.96 ± 1.33	0.78 ± 0.05	4.83 ± 0.46	39.66 ± 0.50	80.09 ± 0.10	0.00 ± 0.00
ETH1	55.89 ± 2.47	50.74 ± 1.48	37.88 ± 1.95	0.75 ± 0.06	4.48 ± 0.73	40.30 ± 0.10	78.07 ± 0.31^**^	11.1 ± 0.66^**^
ETH2	55.34 ± 7.94	49.53 ± 1.98	38.90 ± 4.14	0.78 ± 0.06	4.25 ± 0.61	40.37 ± 0.35	77.38 ± 0.20^**^	28.4 ± 0.88^**^
ETH3	53.09 ± 3.23	53.11 ± 0.74	40.98 ± 1.06	0.77 ± 0.03	5.02 ± 0.43	39.49 ± 0.52	77.04 ± 0.08^**^	46.6 ± 0.19^**^

Data are represented as the mean values ± standard deviation (SD, n=15). ETH1, ETH2, and ETH3 represent 0.5, 1.0, and 1.5 g L^-1^ ethephon, respectively. Double asterisks indicate statistically significant differences at P < 0.01.

We also investigated the growth performance of *C. oleifera* trees under higher ethephon treatments (ETH3, ETH4, and ETH5). The leaf chlorophyll and carotenoid contents significantly decreased when sprayed with 2.0 (ETH4) and 2.5 g L^-1^ (ETH5) ethephon. However, there were no significant differences in the contents of chlorophyll b, chlorophyll ab, and carotenoid in leaves were observed after spraying with 1.5 g L^-1^ (ETH3) ethephon (**Figure S1B**). These results further confirmed that ethephon treatment with no more than 1.5 g L^-1^ would not affect the growth of *C. oleferia*.

### The shell cracking rates were increased in *C. oleifera* after ethephon treatment

Shell cracking is conducive to postharvest processing of *C. oleifera* (Xia and Yao, 2019). We found the shell cracking rates of ETH1, ETH2, and ETH3 of ‘Huashuo’ were significantly higher than the control (**Figures 2A, B, D, E**; **Table 3**). The higher ethephon concentration can obtain a higher cracking rate. When compared with the control, the shell cracking rates of ETH1, ETH2, and ETH3 were 11.1%, 28.4%, and 46.6% of ‘Huashuo’ (**Table 3**). The much obvious phenotypes were observed from the higher ethephon concentrations treatments (ETH4, and ETH5) of ‘Xianglin 210’ (**Figure S2**). But as almost of the shell of seeds had cracked, we did not count the exact values. These findings suggested that the ethephon treatment before fruit ripening also can promote the shell cracking of *C. oleferia*.

The shell structure of unsplit ‘Huashuo’ fruit after ethephon treatment was investigated by SEM. In the fruits of un-dehiscence of the control, the morphology of fiber cells, stone cells and parenchyma cells in the shells of the ventral suture area was intact (**Figure 2C**). However, the shell treated by ethephon showed a distinct dehiscence in the ventral suture area, and the fiber cells in the dehiscence area were broken and lost their original basic shape even the fruits was still showed un-dehiscence (**Figure 2F**). Only part of the parenchyma cells showed abnormal enlargement, probably because they were located at the beginning of the dehiscence. These suggested that the ethephon treatment have accelerated the shell cracking from the inside to the outside than the control.

### Variations in the contents of ACC and sugar in ‘Huashuo’ seeds

To investigate the effect of application of ethephon to the endogenous ethylene of seeds, we measured the ACC contents of seeds under different ethephon concentrations. The data showed that the ACC contents in seeds of ETH1, ETH2, and ETH3 increased by 17.1%, 23.9% and 44.2%, respectively, compared with the control in 2020 (**Figure 1B**). The effects of application of ethephon to the sucrose, soluble sugar, and reducing sugar contents were also analyzed. The results showed that the measured sucrose, soluble sugar and reducing sugar contents were significantly increased under all three ethephon treatments when compared with the control (**Figure 1C**). Similar results were also obtained from the 2021 experiment (**Figure 1D**). These data showed that the application of ethephon clearly promoted the accumulation of ACC and sugar.

### ETH-regulated DEGs identified by transcriptome analysis

We compared the transcriptome of ‘Huashuo’ shells between the control and ETH3 in 2020. Principal component analysis (PCA) showed that the replicates of each treatment were clustered into the same group (**Figure S3**). A total of 99,511 unigenes were generated, and 49,584 unigenes were annotated by at least one database (Nr, Swiss-port, KOG and KEGG) (**Tables S4**, **S5**). The total number of DEGs identified was 6498, including 2938 up-regulated and 3560 down-regulated unigenes. The GO enrichment analysis showed that the top three enriched GO terms for the biological process (BP) category were metabolic process, cellular process, single-organism process; for the cellular component (CC) category were membrane, membrane part, cell, and for the molecular function (MF) category were binding, catalytic activity and transporter activity (**Tables S6**). KEGG analysis showed that most of DEGs were enriched in pathways including LA and ALA metabolism, plant hormone signal transduction, carbon metabolism, and starch and sucrose metabolism (**Figure S4**; **Table S7**). The top 20 KEGG pathways with the highest number of up-regulated and down-regulated DEGs are listed in **Figure S4**. Plant–pathogen interaction (ko04626), plant hormone signal transduction (ko04075), MAPK signaling pathway–plant (ko04016), phenylpropanoid biosynthesis (ko00940), and starch and sucrose metabolism (ko00500) pathways were significantly enriched with upregulated DEGs. The pathways of plant hormone signal transduction (ko04075), starch and sucrose metabolism (ko00500), photosynthesis (ko00195), other glycan degradation (ko00511), and glutathione metabolism (ko00480) were enriched with downregulated DEGs.

**Figure 3:**
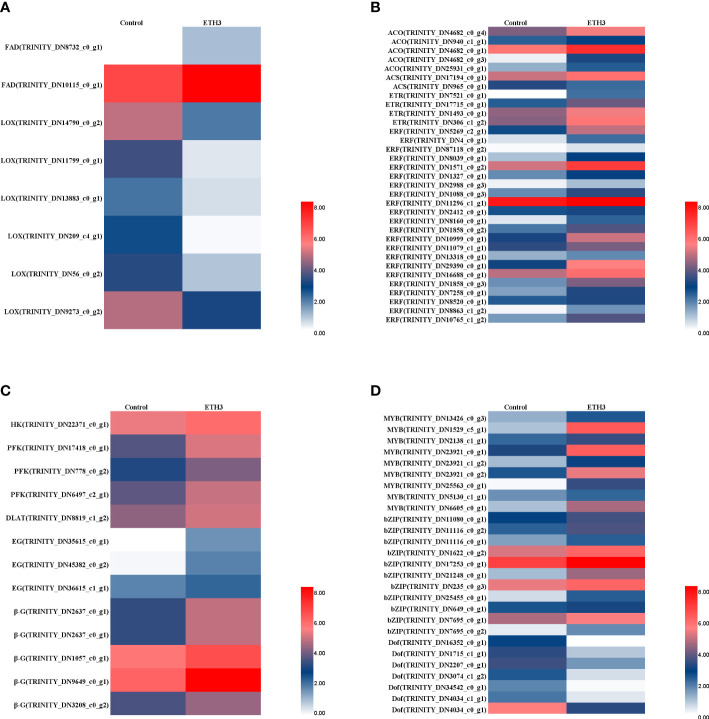
Comparison of expression patterns of key genes between the control and ETH3. **(A)** DEGs related to α-linolenic acid metabolism; **(B)** DEGs related to ethylene biosynthesis and signal transduction; **(C)** DEGs related to Glycolysis/Gluconeogenesis; **(D)** DEGs related to transcription factors. The control and ETH3 indicate the 0 and 1.5 g L^-1^ ethephon, respectively. Enzyme and chemical names are abbreviated as follows; MYB: v-myb avian myeloblastosis viral oncogene homolog; bZIP, basic leucine zipper; Dof, DNA binding with one finger; FAD, fatty acid desaturase; LOX, lipoxygenase; ACO, 1-Aminocyclopropane-1-Carboxylic Acid Oxidase; ACS, acyl-CoA synthetase; ETR, ethylene receptor; ERF, ethylene response factor; HK, hexokinase; PFK, 6-phosphofructokinase; DLAT, dihydrolipoyllysine-residue acetyltransferase; EG, endoglucanase; β-G, beta-glucosidase.

The DEGs involved in the LA and ALA metabolism which include the *FAD* and *LOX* were significantly regulated by ethephon treatment (**Figure 3A**). However, the two *FAD2* were significantly up-regulated, but the six *LOX* genes were significantly down-regulated (**Figure 3A**). These results indicated that ethephon treatment inhibited the conversion of ALA to jasmonic acid, reduced the consumption of ALA, and increased the content in consequence. Many DEGs which are related to ethylene biosynthesis and signal transport-related DEGs, such as ACC synthase (*ACS*), ACC oxidase (*ACO*), and ethylene response factor (*ERF*), showed higher expression levels in ETH3 than the control (**Figure 3B**). The DEGs involved in the starch and sucrose metabolism, such as hexokinase (*HK*), 6-phosphofructokinase (*PFK*), and dihydrolipoyllysine-residue acetyltransferase (*DLAT*) were also up-regulated (**Figure 3C**). These results indicated that ethylene plays an important role in the regulation of carbohydrate metabolism. All eight DEGs in cellulose degradation-related genes, and three endoglucanase (*EG*) and five beta-glucosidase (*β-G*) genes which might be responsible of the shell cracking were significantly up-regulated by ethephon treatment. Besides, a series of transcriptional factors (TFs) related to oil synthesis were up-regulated (*MYB* and *bZIP*) and down-regulated (*Dof*) in response to ethephon treatment (**Figure 3D**).

### Validation of RNA-seq results by qRT-PCR

**Figure 4:**
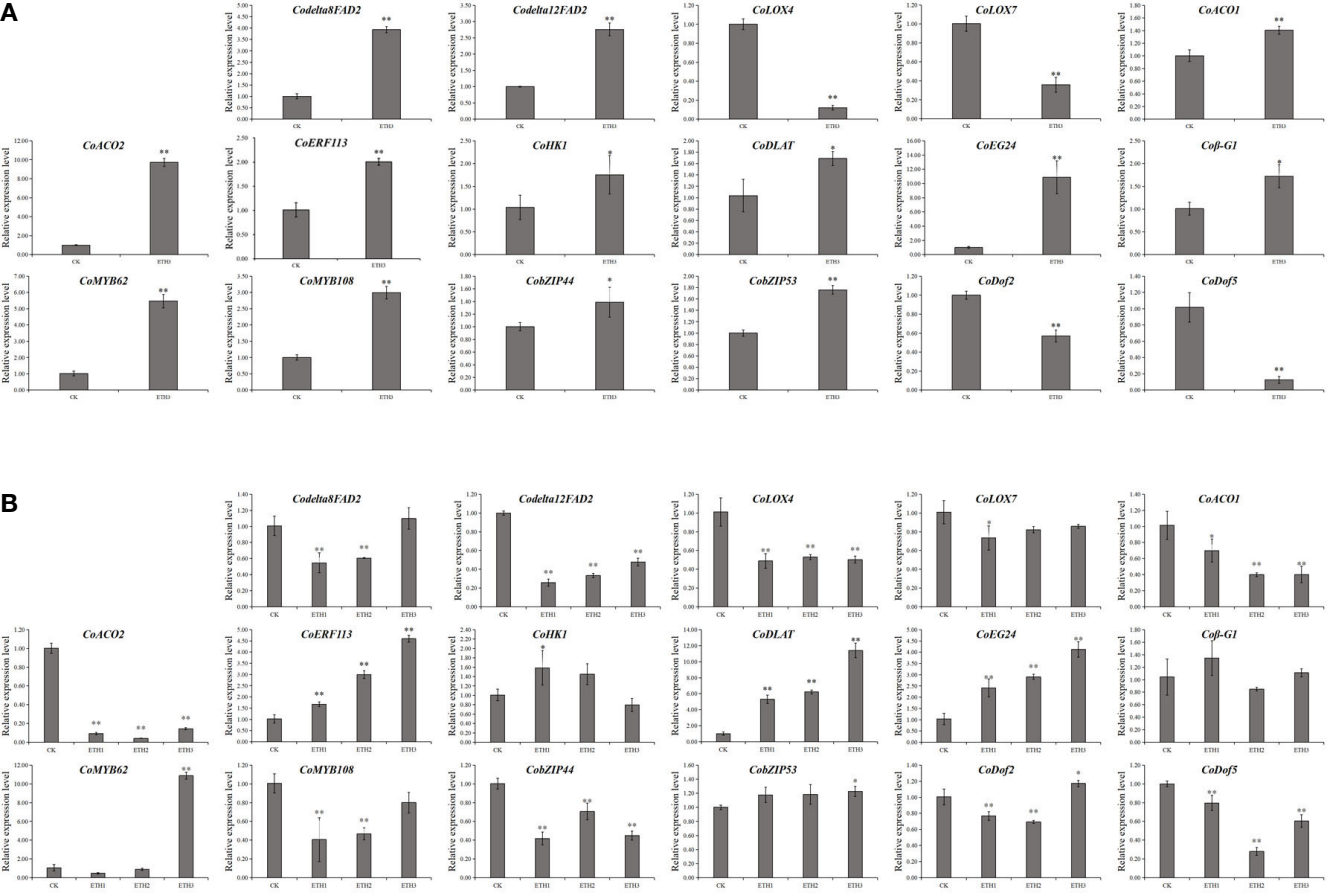
Expression of 17 DEGs identified to LA and ALA accumulation by qRT-PCR analyses from the ethephom treatment. **(A)** Expression levels of DEGs in shells of the control and ETH3 in 2020; **(B)** Expression levels of DEGs in seeds with three ethephon treatments (ETH1, ETH2, and ETH3), and the control in 2020. The control, ETH1, ETH2, and ETH3 represent 0, 0.5, 1.0, and 1.5 g L^-1^ ethephon, respectively. Single and double asterisks indicate differences at *P* < 0.05 and *P* < 0.01, respectively.

Seventeen genes related to LA and ALA synthesis and regulation were selected for qRT-PCR analysis to confirm the RNA-seq results. The results showed that the expression levels of the 17 genes were consistent between qRT-PCR and RNA-seq experiments in shells (**Figure 4A**). To further identify the genes which closely related to the synthesis and regulation of LA and ALA in *C. oleifera* seeds, the expression levels of 17 genes in seeds under different ethephon treatments (ETH1, ETH2, ETH3), and the control in 2020 were further investigated. The results showed that the *Codelta12FAD2*, *CoLOX4*, *CoACO1*, *CoACO2*, *CobZIP44* and *CoDof5* were significantly down-regulated in all three ethephon treatments when compared with the control, while the *CoERF113*, *CoDLAT*, and *CoEG24* were significantly up-regulated in three treatments (**Figure 4B**).

## Discussion

### Ethylene increased the contents of LA and ALA in *C. oleferia*

Previous studies have shown the importance of LA and ALA in the quality improvement of plant oil (Peng et al., 2020; Yang et al., 2020). However, studies on the regulation of LA and ALA synthesis and their molecular mechanism by exogenous application of plant growth regulators are few. In this study, the relative fatty acid contents in *C. oleferia* seeds treated with exogenous ethylene (ethephon) and ethylene inhibitor (AOAA) were determined. We found the LA and ALA contents increased in ethephon treatment, and declined in AOAA treatment. The effect of ethephon on LA and ALA contents was further judged *via* the treatment experiments of different *C. oleferia* cultivars with different concentrations of ethephon. Ethylene increased the contents of LA and ALA. The quality of *C. oleferia* could be improved by ethephon treatment.

Studies have documented that the production of LA and ALA in seed oil is mainly controlled by several *FAD* genes, such as *FAD2* (Dar et al., 2017). *FAD2* gene mutations significantly alter fatty acid profiles in peanut (Wang et al., 2015). *LOX* plays an important role in the conversion of LA into other substances (Nan et al., 2014). In our study, we also found the genes involved in the LA and ALA metabolism, such as *CoLOX4*, showed lower expressions in the shells and seeds of ethephon treatment (**Figure 4**). Besides, *ERFs* are the response genes of the ethylene signaling pathway and could regulate plant development processes by binding to the promoters (Gu et al., 2017; Hao et al., 2018). Our results showed that the expressions of gene *CoERF113* which are involved in ethylene biosynthesis and signal transduction was up-regulated under ethephon treatment in shells and seeds (**Figure 4**). In addition, *DLAT* plays an important catalytic role in the conversion of pyruvate to acetyl-CoA, which is the starting material of lipid synthesis (Patel et al., 2014). We found that *CoDLAT* was also up-regulated under ethephon treatment in shells and seeds (**Figure 4**). Combining with the change patterns of unsaturated fatty acids, ACC and sugar contents, the *CoLOX4*, *CoERF113*, and *CoDLAT* might play important roles in LA and ALA synthesis and their regulation.

It was reported that the increased oil content, fatty acid content, and flux of glycolysis has great consistent in *Brassica napus* (Schwender et al., 2015). Sucrose was confirmed to play a key role in sugar metabolism. The sucrose content increases with the triacylglycerols accumulation in potato tubers (Hofvander et al., 2016), and the content is also closely related to oil synthesis (Hernández et al., 2012; Song et al., 2021). In addition, the contents of soluble sugar and reducing sugar were proved positively correlated with the LA and ALA contents (Zhang et al., 2021). Our results also found the exogenous ethephon treatment could coordinate increase the sucrose, soluble sugar and reducing sugar with the accumulations of LA and ALA in *C. oleifera* (**Figures 1C, D**). Together, these suggested that the genes and metabolic products of sugar metabolism were highly related with the accumulations of LA and ALA.

### Ethylene promoted the shell cracking of *C. oleifera*

A series of studies has reported that ethylene is the main signal molecule to regulate fruit ripening and abscission in plants (Roongsattham et al., 2012; Vandenbussche et al., 2012; Xin et al., 2019). Ethylene is an important regulator of climacteric fruit ripening (Alexander and Grierson, 2002; Al-Saif et al., 2017). Ethylene also can enhance fruit cracking. Chen et al. (2019) showed that ethylene treatment of African Pride atemoya can accelerate the fruit ripening and cracking after harvest. The shell of postharvest *C. oleifera* is mainly peeled by sun exposure until the shell cracks and then manually removed. This method of waiting for the shells to crack is not only inefficient but also a waste of labor and time (Xia and Yao, 2019). To save the time consuming on the shelling of *C. oleifera*, mostly focuses on mechanization at present (Xia and Yao, 2019). In this study, we found that the ethephon has positive effect on fruit cracking of *C. oleifera* with a dosage dependent (**Table 3**). However, the higher ethephon concentrations have adverse effects to normal growth of *C. oleifera* (**Figure S2**). We further found the 1.5 g L^-1^ ethephon treatment one week before harvest not only could significantly promote the shell cracking of *C. oleifera* (**Table 3**), but also with no significant differences in the leaf growth and fruit phenotypic traits (**Figure S1**; **Table 3**). This provides a method to promote the shell cracking of *C. oleifera* in an efficient way which can be used in tea oil extraction.

## Data availability statement

The original contributions presented in the study are publicly available. This data can be found here: https://www.ncbi.nlm.nih.gov/sra/PRJNA899242.

## Author contributions

XM and DY designed the research. HL mainly performed the research. WW, JZ, and YL finished some parts of the experiments. XM wrote the manuscript. XM, HL, and DY revised and approved the manuscript. All authors contributed to the article and approved the submitted version.

## Supplementary material

Supplementary Figure 1 Contents of chlorophyll and carotenoid in leaves with different treatments on *C. oleifera* cultivars ‘Huashuo’ **(A)** and ‘Xianglin 210’ **(B)**. ETH1, ETH2, ETH3, ETH4, and ETH5 represent 0.5, 1.0, 1.5, 2.0, and 2.5 g L^-1^ ethephon, respectively. Single and double asterisks indicate differences at *P* < 0.05 and *P* < 0.01, respectively.

Supplementary Figure 2 Shell cracking of *C. oleifera* ‘Xianglin 210’ treated with different concentrations of ethephon. The control **(A)**, ETH3 **(B)**, ETH4 **(C)**, and ETH5 **(D)** represent 0, 1.5, 2.0, and 2.5 g L^-1^ ethephon, respectively.

Supplementary Figure 3 Principal component analysis of ETH3 and the control. ETH3 indicates the 1.5 g L^-1^ ethephon.

Supplementary Figure 4 Enrichment analyses of up-regulated **(A)** and down-regulated **(B)** differentially expressed genes (DEGs) between the control and ETH3. The control and ETH3 indicate the 0 and 1.5 g L^-1^ ethephon, respectively.
