# Supplementary figures and images for: Enhancing the accumulation of linoleic acid and α-linolenic acid through the pre-harvest ethylene treatment in Camellia oleifera
Source: Front Plant Sci. 2023 Feb 24;14:1080946. doi: 10.3389/fpls.2023.1080946 (PMC9999010; doi:10.3389/fpls.2023.1080946)

Figure S1

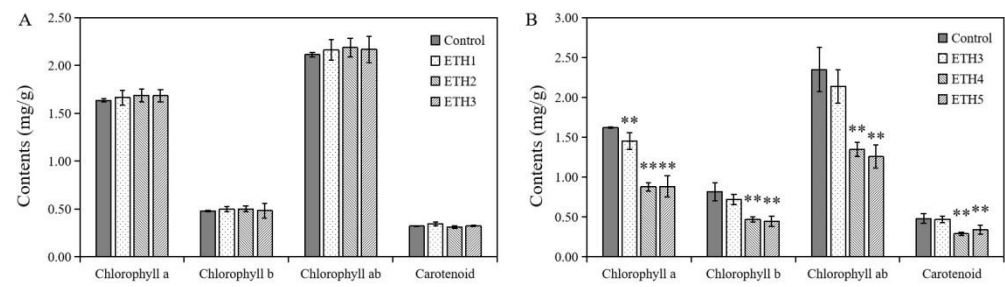

Figure S2

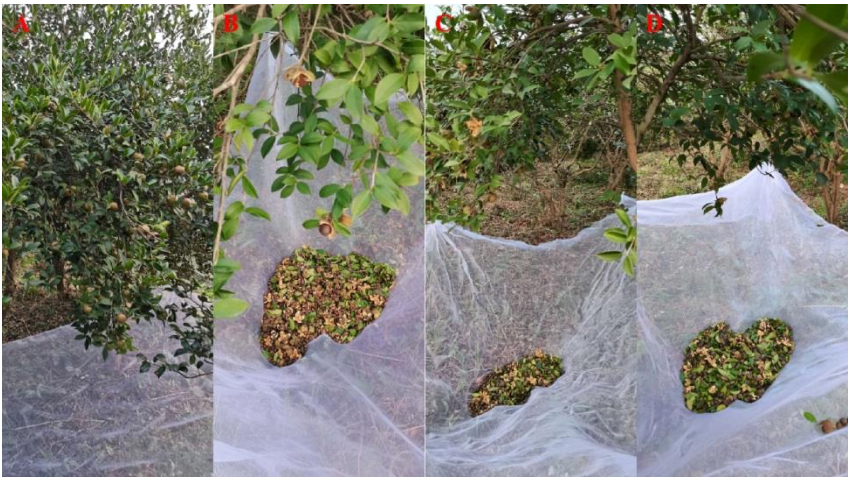

Figure S3

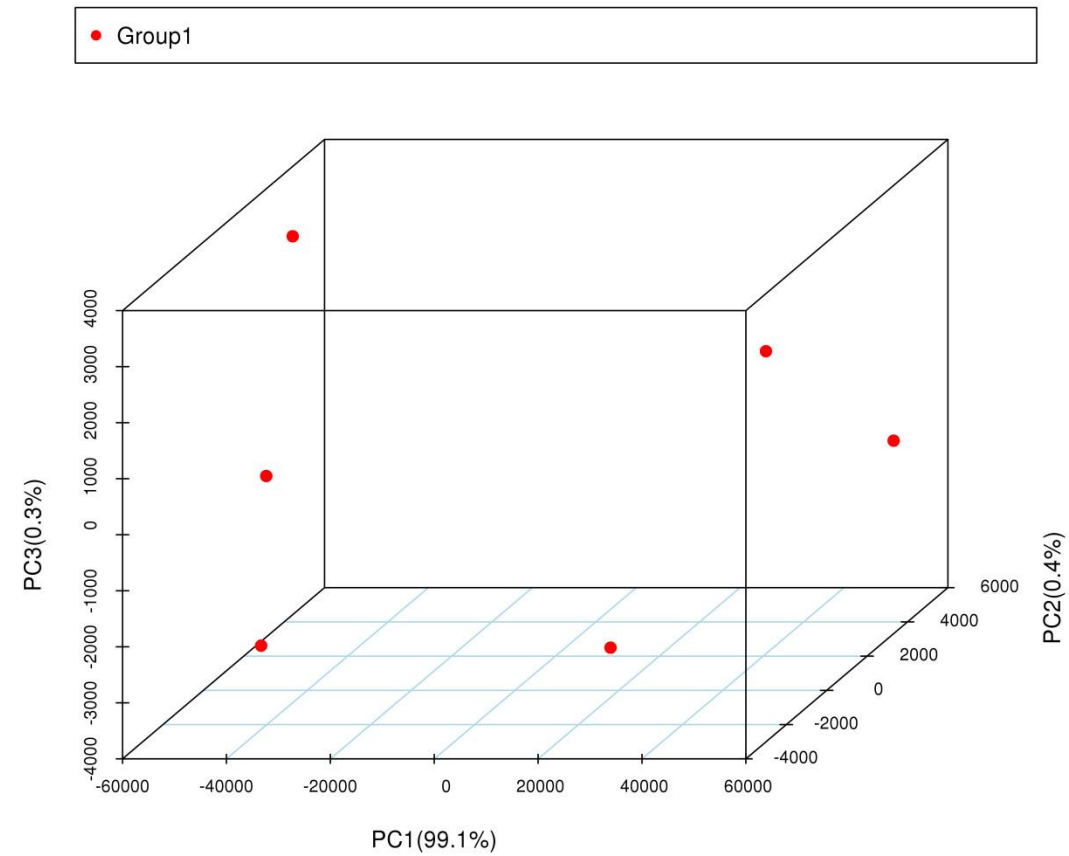

Figure S4

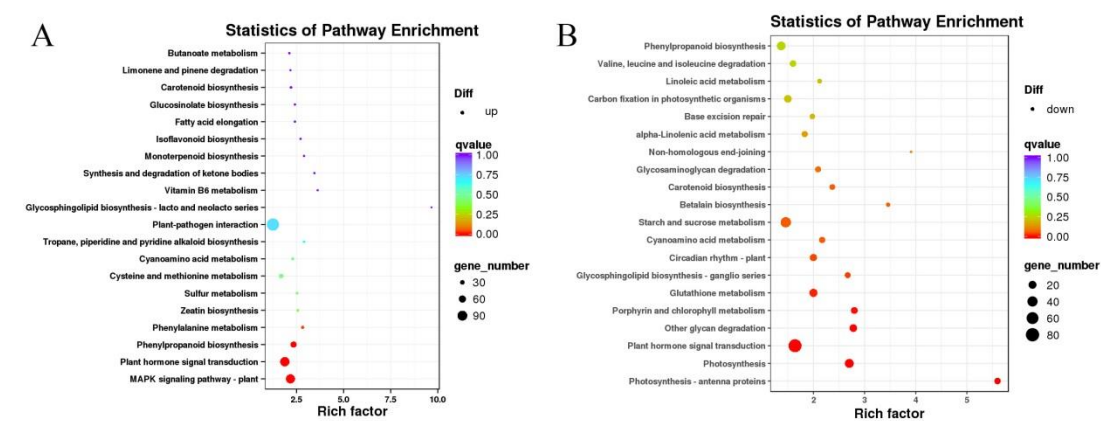

Supplement: Supplementary Figure 1 — Contents of chlorophyll and carotenoid in leaves with different treatments on C. oleifera cultivars ‘Huashuo’ (A) and ‘Xianglin 210’ (B). ETH1, ETH2, ETH3, ETH4, and ETH5 represent 0.5, 1.0, 1.5, 2.0, and 2.5 g L-1 ethephon, respectively. Single and double asterisks indicate differences at P < 0.05 and P < 0.01, respectively. [file DataSheet_1.pdf]
